# Dielectrophoresis Manipulation: Versatile Lateral and Vertical Mechanisms

**DOI:** 10.3390/bios9010030

**Published:** 2019-02-26

**Authors:** Muhamad Ramdzan Buyong, Aminuddin Ahmad Kayani, Azrul Azlan Hamzah, Burhanuddin Yeop Majlis

**Affiliations:** 1 Institute of Microengineering and Nanoelectronics (IMEN), Universiti Kebangsaan Malaysia (UKM), Bangi, Selangor 43600, Malaysia; azlanhamzah@ukm.edu.my (A.A.H.); burhan@ukm.edu.my (B.Y.M.); 2 Functional Materials and Microsystems Research Group, School of Engineering, RMIT University, Melbourne, VIC 3001, Australia; aminuddin.kayani@staff.mmu.edu.my; 3 Centre for Advanced Materials and Green Technology, Multimedia University, Melaka 75450, Malaysia

**Keywords:** dielectrophoresis, lateral and vertical manipulation

## Abstract

Discussing the topic of the capability of dielectrophoresis (DEP) devices in terms of the selective detection and rapid manipulation of particles based on the DEP force (F_DEP_) via contactless methods is challenging in medical research, drug discovery and delivery. Nonetheless, the process of the selective detection and rapid manipulation of particles via contactless DEP based on dielectric particles and the surrounding medium can reduce the effects of major issues, including physical contact with the particles and medium contamination to overcome operational difficulties. In this review, DEP microelectromechanical system (MEMS) microelectrodes with a tapered profile for the selective detection and rapid manipulation of particles were studied and compared with those of conventional designs with a straight-cut profile. The main objective of this manuscript is to review the versatile mechanism of tapered DEP MEMS microelectrodes for the purpose of selective detection and rapid manipulation. Thus, this review provides a versatile filtration mechanism with the potential for a glomerular-based membrane in an artificial kidneys’ development solution for implementing engineered particles and cells by lateral attraction as well as vertical repulsion in the development of lab-on-a-chip applications. For tapered DEP MEMS microelectrodes, the scope of this study methodology involved the characterisation of DEP, modelling of the polarisation factor and the dynamic dielectric changes between the particles and medium. Comprehensive discussions are presented on the capability of tapered DEP microelectrodes to drive the selected particles and the simulation, fabrication and testing of the tapered profile. This study revealed an outstanding performance with the capability of producing two regions of high electric field intensity at the bottom and top edges of the side wall of tapered microelectrodes. Observations on particle separation mainly by the lateral attraction force of particles with positive DEP on the y-axis and vertical repulsion force of particles with negative DEP on the z-axis proved an efficient and uniform F_DEP_ produced by tapered electrodes. In conclusion, this study confirmed the reliability and efficiency of the tapered DEP microelectrodes in the process of selective detection and rapid manipulation at a higher efficiency rate than straight-cut microelectrodes, which is significant in DEP technology applications.

## 1. Introduction

Each particle has its own physical and chemical properties. The uniqueness of these parameters can be used for the separation and manipulation process in mixing different types of particles using the dielectrophoresis (DEP) technique. In natural occurrences, this can be observed in the inseparable boundary between fresh and saltwater particles between the Mediterranean and the Atlantic Ocean in the Gibraltar. These particles can still be differentiated since each particle has its own distinctiveness. Thus, this review describes and discusses the versatile mechanism only by introducing an alternative method of both capabilities, namely lateral attraction at the y-axis and vertical repulsion at the z-axis. Manipulation of particle polarisation is based on the unique properties of the particles for identification. This allows versatile mechanism manipulation and separation processes to be performed for mixing various types of particles in a liquid medium.

Today, the process of manipulating and separating particles is divided into two main techniques, which are contact and contactless techniques. For instance, particle manipulation and separation processes using the contact technique include pressure-driven membrane filtration using a micro/nano membrane [1], a microgripper [2], label/marker techniques such as epithelial cell adhesion molecule (EpCAM), fluorescence-activated cell sorting (FACS) and the mixing of magnetic materials including magnetic activated cell sorting (MACS) [3]. In general, direct contact techniques have a negative impact on particles and their medium environment. More important are the physical and chemical damages experienced by the particles and the medium contamination due to direct contact and the addition of contamination materials, including marker and magnetization, which can influence the effectiveness of manipulation and separation. Consequently, the main issue affecting pressure-driven membrane filtration is the physical contact of the target particle surface and vice versa, which is similar to the addition of a marker and magnetisation techniques. Therefore, the solution to the problem of manipulation and separation of the proposed label-free, contactless technique particles is the use of DEP technology [4,5,6,7,8,9,10,11,12,13].

The development of DEP’s field of research focusing on the use of particle manipulation and separation processes has been strongly emphasised by world-renowned scientists through the techniques of contactless solution [14,15,16,17,18]. The use of DEP technology enables the process of particle manipulation and separation to be performed using dielectrophoresis forces (F_DEP_) based on the dielectric value between the particles and medium environment. This is because particle movement, positioning and stationing for manipulation and separation purposes are crucial if additional marking materials are not present. There are many advantages that can be gained from the use of DEP fields. For instance, the target and non-target particles and environment in the medium are not affected either physically or chemically during the DEP procedure. In addition, they can reduce and eliminate the negative impacts on the particles and medium contamination. Operational complexity levels can be simplified, which will reduce operating costs. Moreover, the efficient manipulation and separation processes at high rates can be gained from DEP techniques.

Advanced application technology for medical use depends on the efficiency of the manipulation and separation processes for mixed groups of cells and biomolecules. At present, DEP’s ability to manipulate and separate cells and biomolecules based on F_DEP_, which is a contactless process method, has been introduced and progressively performed in medical studies. This is because the electrokinetic DEP application has the potential to be used as a MEMS sensor and actuator for detection and manipulation. Specifically, this involves the process of detection, enumeration, focusing and separation of target cells and biomolecules. The ability of DEP to choose one type of cell and biomolecule can benefit the development of an artificial kidney. The separation process between good cells and unwanted toxins can be performed during the blood cleaning process known as haemodialysis. 

**Figure 1:**
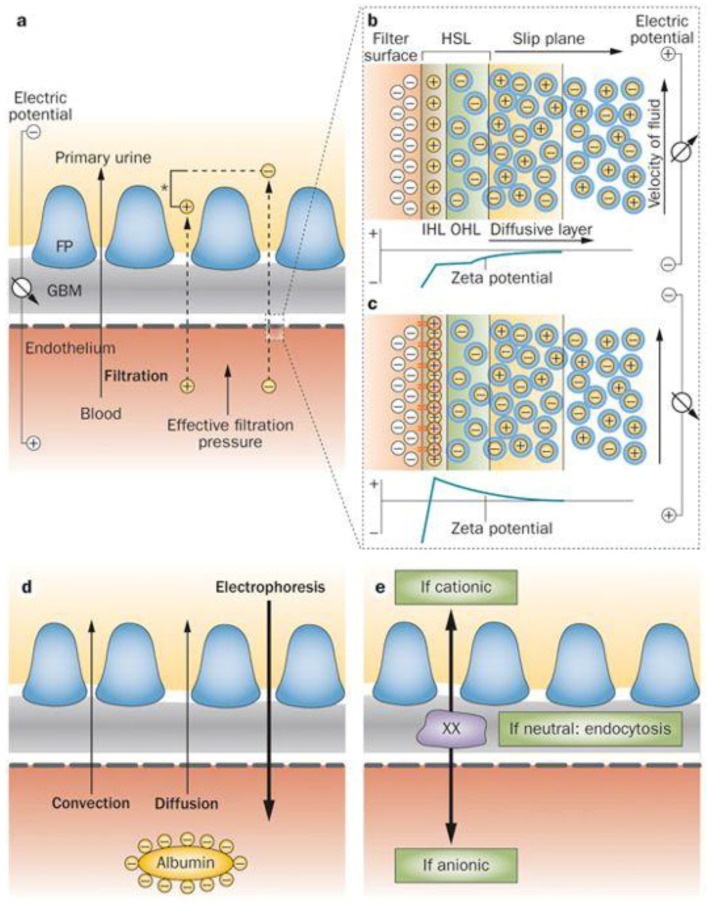
The human body waste filtration at a glomerular-based membrane: (**a**) the ultrafiltration in a glomerular-based membrane consisted of a two-pore model (contact) and electro-kinetic model (contactless), (**b,c**) the electrical model for an active filtration by electrophoresis, (**d**) the mechanism of passive filtration by convection, diffusion and active filtration by electrophoresis and (**e**) the anti-clogging for selective detection and rapid manipulation [19].

Potential DEP implementation is still under investigation for human organs-on-chip (CoC) application, for example, in imitating the human kidneys. The task of the kidney is to sustain a healthy body and well-being. The kidneys’ main function is in the urinary organ where filtration, reabsorption and secretion occur. In this regard, we use DEP as a glomerular filtration mechanism, which is a critical process of the human kidney which ensures that only waste and excess water are removed from the body. These processes can be explained by blood cleaning through glomerular-based membrane filtration with two different mechanisms as shown in Figure 1. First, the pore model filtration based on the diffuse and convention process is shown in Figure 1a,d. This technique is similar to a human-made haemodialysis dialyser based on the passive filtration method. Second is the electrokinetic model filtration based on selective detection and rapid manipulation as shown in Figure 1b,c,e. The implementation of DEP in selective detection is based on the target particles of their dielectric properties. The dielectric properties are derived from physical and chemical properties by target particles. Consequently, different transitions of DEP working frequencies are subjected to their own dielectric properties. The rapid manipulation is based on differences in the dielectric properties of target and non-target particles. Therefore, different DEP working frequencies are applied to target particles and non-target particles under DEP occurrence. This results in the manipulation of target particles and non-target particles at the inner and outer regions of interest, respectively. Thus, this requirement provides a versatile filtration with potential for a glomerular-based membrane in an artificial kidney development solution.

The contactless process of manipulating and separating particles through the DEP method is based on the dielectric value of the particles and medium environment [20,21,22,23,24,25] subject to the input frequency applied, which generates two pole microelectrodes. The early stage of development in the DEP theory involves wire electrodes. However, to date, most researchers use microfabrication methods for patterning microelectrodes. The integration of CMOS and hybrid micro- to nanofabrication technology proves the versatile DEP capability for lateral or vertical manipulation and separation. 

Tapered DEP microelectrodes facilitate efficient manipulation and separation processes. In previous works involving DEP, the force drive generated using a straight-cut profile of DEP microelectrodes, which results in lateral or vertical DEP driving forces only, clarifies only the lateral or vertical for the manipulation and separation of alignments at different magnitudes at the similar axis. A problem occurs in the mixing of two or more mixtures containing different particle sizes and types, in particular, particles collected at a similar axis but have different magnitude of strengths. One of the latest works of tapered DEP microelectrodes for the efficient manipulation and separation of particles was studied and compared with straight-cut profile of DEP microelectrodes. This was due to its ability to perform selective manipulation and separation processes using polystyrene (Ps) engineered particles and biological cells, red blood cells and platelets. Polarisation factor modelling and dielectric properties change between particles explain the ability of tapered DEP microelectrodes to select target particles. 

In order to determine the F_DEP_ for an electrostatic or quasi-static applied field due to the translational force experienced, the parameters of the intrinsic dielectric properties of the particles and medium need to be understood. The reaction of the dynamic dielectric properties of the particles and medium-induced dipole moments are aligned parallel to or against the high intensity electric field sources. This interaction results in the transformation of different and unique dielectric polarisation factors of particles suspended in liquid medium when exposed to the non-uniform electric field ∇*E*. Furthermore, utilising tapered DEP microelectrodes to generate two spots of higher intensity electric field allows different types of particle detection and lateral and vertical manipulation applications to be conducted. Therefore, the fine-tuning of alternating current (AC) input frequency is selected as the control parameter. Consequently, the connection of dynamic dielectric properties of particles, medium and electric field are abbreviated using the Clausius–Mossotti factor (CMF) in F_DEP_ formulation as in Equation (1):

(1) FDEP=2 π εo εmedium r3 Re CMF ∇E2

where *ε_o_ε_m_* is the absolute permittivity of the suspending medium, *ε_o_* is the permittivity for vacuum 8.854 × 10^−12^ F/m and *ε_m_* is the relative permittivity of the suspending medium. The induced dipole moment is a ponderomotive effect interrelated to the particle volume, *r*^3^. For larger particles, a higher magnitude of F_DEP_ is experienced while the CMF represents the effective electrical polarisability due to the dynamic dielectric properties of the particle in relation to the surrounding of the medium. The frequency dependent reaction is formulated by

(2) $$\mathrm{CMF}=\frac{\left(\varepsilon^{*}{}_{\mathrm{particle}\;}-\varepsilon^{*}{}_{\mathrm{medium}\;}\right)}{\;\left(\varepsilon^{*}{}_{\mathrm{particle}\;}+2\;\varepsilon^{*}{}_{\mathrm{medium}}\right)}$$

where

(3) $$\varepsilon^{*}{}_{\mathrm{particle}\;}=\varepsilon_{\mathrm{particle}}-\frac{j\sigma_{\mathrm{particle}}}{\omega }$$

and

(4) $$\varepsilon^{*}{}_{\mathrm{medium}}=\varepsilon_{\mathrm{medium}}-\frac{j\sigma_{\mathrm{medium}}}{\omega }$$

The *ε*_particle_ represents the absolute permittivity of the particle, *σ*_particle_ is the conductivity of the particle and σ_medium_ represents the conductivity of the medium. The polarisation factor efficiency of CMF varies from 1 to 0 for positive DEP forces (P_DEP_) and from 0 to −0.5 for negative DEP forces (N_DEP_). At a CMF of 0, the P_DEP_ and N_DEP_ value forces are equal to static conditions known as crossover frequency (*fxo*) for each type of particles. The CMF value is formulated based on the intrinsic dielectric properties such as the permittivity and conductivity of the particles and medium subjected to the input frequencies applied of the AC electric field. The implementation of tapered DEP microelectrodes proliferates the non-uniformity of the electric gradient. In consequence, the two spots of higher intensity electric field enable the enhancement of the sensitivity and selectivity of detection with lateral and vertical manipulation capabilities. 

The two main challenges in applying DEP involves (i) a high medium conductivity (normally in biological application) and (ii) a higher magnitude of electric fields due to the miniaturisation of DEP microelectrodes. This creates an electrothermal motion in the aqueous solution which is also known as the Joule heating effect [26,27,28]. This can be assumed to be the advantage of tapered DEP microelectrodes where dominant DEP forces generated eliminate the need for splitting one spot of intensity electric field into two spots of intensity electric fields. The reduced magnitude electric field is exposed to medium and suspended particles. In parallel, reducing a higher magnitude electric field application enables the minimisation of issues in the electrohydrodynamic force of a higher medium conductivity. This results in the DEP force appearing to be dominant, followed by electrothermal flows that affect the electrohydrodynamic force in DEP.

## 2. Prior Art Dielectrophoresis

In this review, the classification of straight-cut DEP microelectrodes configurations is summarised. They are divided into 12 different configurations [29]. Within each type, introduction techniques are classified based on their working mechanisms with their advantages and limitations discussed. Different configurations are made based on current fabrication technologies, as a solution to existing problems, and to meet innovation and application requirements. The classification of the straight-cut profile DEP microelectrodes configuration are as follows:

1. *Interdigitated*

The layout and design of this type of microelectrode are parallel between the two poles of adjacent bar microelectrodes. Today, interdigitated configurations are widely used in DEP’s core investigations since they can be easily fabricated with many existing references. In fact, interdigitated microelectrodes are the initial development of the DEP microelectrodes miniaturisation process after the development of the DEP concept that uses wire microelectrodes.

2. *Castellated*

The layout and design of this type of microelectrode are opposite to the two polar-shaped microelectrodes poles. Similar to the interdigitated, the castellated has been also greatly developed. There is no significant difference between the interdigitated and castellated. However, the DEP response for the interdigitated is adjacent to the two microelectrodes. In contrast to the castellated, the DEP response is the opposite of two microelectrodes. 

3. *Oblique*

The layout and design of this type of microelectrode are adjacent to the two polar bars. The straight bar microelectrode pair has a larger opening prefix and tends to be the end of a smaller opening distance. Oblique microelectrodes are suitable for the continuous flow separation of particles in the microfluidic level. Consequently, the configuration of the oblique electrode has been developed for DEP fluid flow fraction (FFF) application. 

4. *Curved*

The layout and design of this type of microelectrode are adjacent to the two pole polar bars like the oblique microelectrodes. The difference is that the curved microelectrode bar has a large prefix and narrows at the end of the openings. In addition to improving the functionality of the oblique electrode, the development of the curved electrode is performed with the capability to enhance the selectivity and sensitivity by producing a high intensity electric field at the end of the electrode tip.

5. *Quadrupole*

This microelectrode is structured quad-rectangular between four different input frequency phases to produce an electric field rotation. The configuration of the quadrupole electrode is used for detailed particle manipulation with droplet sample application. Different frequency input phases rotate the particles’ orientation by changing the input frequency phase supplied on the quadrupole electrode.

6. *Microwell*

The layout and design of this type of microelectrode are round circular microelectrodes like donuts. The microwell electrode configuration is used to trap target particles in circular electrodes with droplet sample applications. Manipulated on different input frequencies applied, the microwell electrode traps the target or non-target particles based on the CMF polarisation factor. 

7. *Matrix*

The layout and design of this type of microelectrode are round or rectangular microelectrodes composed by adjacent microelectrodes in opposite poles. The matrix electrode configuration is utilised to trap the target or non-target particles on the top surface of the electrode under P_DEP_ forces. This is different for the interdigitated, castellated, oblique and curved microelectrodes in which the DEP response changes the position away from the electrode under N_DEP_ force. 

8. *Extruded*

This microelectrode is round or rectangular with an adjacent polar microelectrode. The separation process of the extruded electrode is performed by combining the DEP force and pillar structure between the two DEP microelectrodes. The target particles are only impressed with the DEP force, otherwise the non-target particles will be trapped between pillar structures. 

9. *Top-bottom patterned*

These are parallel microelectrodes between two poles of the adjacent bar microelectrodes that intertwine polarities with each other. These electrodes are patterned in the upper and lower position. It enhances DEP forces exposed to target and non-target particles but with a complex fabrication process. 

10. *Sidewall patterned*

The layout and design of this type of microelectrode are built on the edge wall of the medium flow channel. The opposite microelectrodes are similar since the poles and adjacent microelectrodes are different poles. The benefit of this configuration is that it is suitable for continuous flow application, particularly for FFF. However, this configuration requires many microelectrodes and long microchannels for separation application. 

11. *Insulator-based or electrodeless*

The layout and design of this type of microelectrode are built on the sides, left and right only. In the middle, there is an insulating material forming the membrane columns that trap the particles from the electric field, resulting in the microelectrodes side of the left and right. This configuration is similar to the extruded microelectrodes using pillars or membrane columns for separation applications. 

12. *Contactless*

These electrodes are built on the edge sidewall patterned microelectrodes. The advantage of this type of DEP electrode lies on the contactless microelectrodes configuration that is not directly exposed to the medium and particles. The DEP forces are exposed to target and non-target particles via a specific microchannel.

The differences in operating strategies are based on the configurations of the microelectrode, which are divided into 8 different strategies. The classification of the operation strategy on the lateral attraction at the y-axis or vertical repulsion of the z-axis of DEP device [29] are as follows:

1. *Lateral sorting at the y-axis*

The operation strategy of the manipulation and separation process is used when the particles are diverted horizontally across channels since the N_DEP_ force is horizontal at the y-axis at different distance magnitude positions. The manipulation and separation processes depend on the magnitude and drag of the medium flow rate using curved microelectrodes. At high velocities of the medium flow, the particles are concentrated in between two microelectrodes. On the other hand, if the velocity of the particles is low, it will be deduced at the edge of microelectrodes.

2. *Electrothermal-assisted at the y-axis*

The operation strategy of this manipulation and separation processes is used when a high conductivity medium is used to impose an electrothermal vortex in the DEP chamber. The P_DEP_ attraction exposure on the y-axis manipulates the particles from the location of the electrodes to the end of the electrodes at the centre of the meeting of four bar microelectrodes. This operation strategy uses the droplet technique to minimise sample usage. 

3. *Multiple frequencies at the y-axis*

The operation strategy of the manipulation and separation processes is used when the particles are exposed to two or more sources of electric fields with different input frequency values between the two adjacent microelectrodes. This strategy allows particles to be exposed to the attraction of P_DEP_ at the y-axis, which is similar to the electrothermal-assisted at the y-axis using the droplet technique to minimise sample usage. 

4. *Gravitational Field Flow Fraction at the z-axis*

The operation strategy for floating particles or levitation particles is based on the N_DEP_ force vertically at the z-axis at different magnitude of altitude positions. The parabolic flow of the medium causes different velocity particles for different masses of particles weight. This operation strategy is the basic and common method in DEP based on interdigitated microelectrodes, which is suitable for continuous flow using an FFF separation. 

5. *Multistep at the z-axis*

In this method, particles are trapped and released through P_DEP_ attraction vertically at the z-axis at different magnitudes of altitude positions. The parabolic flow of the medium causes particles to move based on radius and polarisation factors. This operation strategy is similar to the gravitational field flow fraction at the z-axis using interdigitated microelectrodes. However, the manipulation input frequency is applied by stepping on “on” and “off”. 

6. *Barrier-assisted at the z-axis*

Particles are rejected and lifted due to the N_DEP_ force vertically on the z-axis. The particles are trapped behind the pillars that interrupt the drag of the medium flow using extruded insulator-based or electrodeless microelectrodes. The integration of interdigitated microelectrodes or end-to-end microelectrodes is suitable for continuous flow of an FFF application. 

7. *Travelling wave at the z-axis*

Wave travel is generated by microelectrodes with quadrature phases of 0°, 90°, 180° and 270°, and particles movement occurs due to the N_DEP_ force exposure vertically on the z-axis against the particles. At the same time, particles are exposed to wave travel that is parallel to the substrate. Different types of particle are directed to different directions. 

8. *Pulsed DEP at the z-axis*

Particles are exposed to F_DEP_ that signals on and off, causing particles to compete with the drag of the medium stream. The particles are pushed forward between two points depending on size. This operation strategy is similar to the gravitational field flow fraction at the z-axis and multistep at the z-axis. The difference is that the N_DEP_ force at the z-axis is used for manipulation and separation processes in the static fluid or droplet technique. 

**Figure 2:**
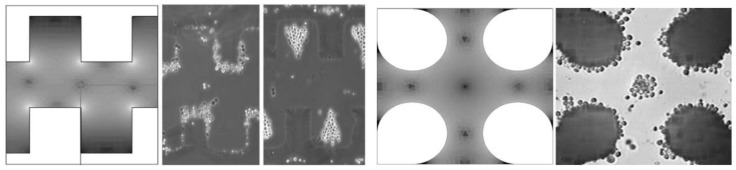
The lateral attraction dielectrophoresis (DEP) [30].

Based on the literature review on the straight-cut DEP microelectrodes, most of the work operating strategies are optimally done by P_DEP_ and N_DEP_ related to the lateral attraction or vertical repulsion. Lateral attraction happens when the particle movement is from a horizontal direction at the y-axis from a low electric field area towards to the top edge of the straight-cut DEP microelectrodes under P_DEP_ conditions. In contrast for N_DEP_, the particles’ movements are vertically directed to the z-axis from a high electric field area towards a low electric field. Figure 2 illustrates the simulation and the experimental results of a lateral attraction at the y-axis movement using the straight-cut DEP microelectrodes of the castellated and polynomial. High intensity electric fields are located on the edge of the microelectrodes. Experiments are performed by exposing P_DEP_ particles at the edges of the microelectrode walls and N_DEP_ in between the two microelectrodes. Based on the simulation and experimental results, high intensity electric field areas produced P_DEP_ that expose the lateral attraction or N_DEP_ exposure lateral repulsion [30,31,32,33]. 

**Figure 3:**
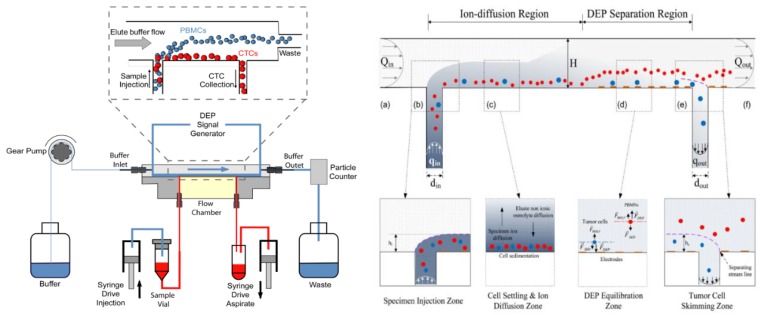
The vertical attraction (left) [34] and repulsion (right) [35,36] DEP.

Vertical movement at the z-axis occurs when particles are vertically attracted toward or repel away from the area edges of a higher intensity electric field of the straight-cut DEP microelectrodes. In Figure 3, the left side shows the evolution of DEP technology led by Ronald Pethig and Peter Gascoyne. Pethig developed a DEP system named ApoStream^TM3^ [34,35,36,37]. The separation ApoStreamTM system application is used as a monitoring system for a metastatic tumour disease known as tumour cell carcinoma. The system works by separating and enumerating the mixture of tumor and normal cells by vertical repulsion, respectively, at the z-axis. Tumors cells are vertically attracted to the microelectrodes, which are accumulated at the surface of microelectrodes. This is in contrast for normal cells that are vertically repelled away from microelectrodes and later accumulated at above the microelectrodes’ surface. Hydrodynamic fluid flow fractionates both cells at the x-axis and are collected at the bottom and middle areas of the fluid flow channel shown in Figure 3. The figure on the right displays a similar configuration of the application based on straight-cut DEP microelectrodes. The N_DEP_ force vertically repels the normal cells, while the P_DEP_ force attracts the tumor cells. The F_DEP_ source is on the upper edges of the microelectrodes. Thus, the separation process occurs at the z-axis only with different magnitude of strengths. The P_DEP_ vertically attracts the tumor cells while the N_DEP_ vertically repels the normal cells. The electric field that builds the F_DEP_ for both systems is a series of the fluid flow path of the medium in the x-axis utilising the hydrodynamic of the parabolic fluid flow fractionated by skimming both cells at the end of the microfluidic channel.

A thorough review involving the lateral attraction or vertical repulsion has reported two different modes, which are lateral attraction or vertical repulsion (Table 1). Several studies in the lateral attraction mode only by the implementation of P_DEP_ and N_DEP_ forces have been completed. These are similar to the vertical repulsion mode by the implementation of P_DEP_ and N_DEP_ forces which results in the manipulation and separation that occurs at one direction of the axis with different magnitudes. This method requires many microelectrode structures and is a time-consuming approach. To the best our knowledge, there is no study available on the combination of lateral attraction and vertical repulsion. This is important to generate two different directions of axis at the y- and z-axes for a better yield of manipulation or separation. Such a study is important for the rapid and selective multifunctional DEP manipulation at two different drive directions and locations by P_DEP_, the lateral attraction at the y-axis to the top surface of microelectrodes, and N_DEP_, the vertical repulsion at the z-axis between two microelectrodes. Subsequently, this study presents perspective developments of the tapered DEP microelectrodes for lateral and vertical manipulation as well as the separation application at two different locations simultaneously.

## 3. Tapered DEP Microelectrodes

The development process of the tapered DEP microelectrode consists of three main phases. The first phase is Finite Element Method (FEM) simulation, which defines the tapered angle value and analyses the optimum angle to produce two spots of high intensity electric field gradient areas. Further analyses are conducted to ascertain the particle trajectory by the lateral and vertical directions. The second phase is to develop a fabrication process of the tapered DEP microelectrodes in a microfluidics channel. Further, the third phase is to obtain the polarisation factor, crossover frequency (*fxo*) and frequency adjustment (*fadj*) of target and non-target particles for manipulation and separation in the lateral and vertical directions. 

### 3.1. FEM Simulation

**Figure 4:**
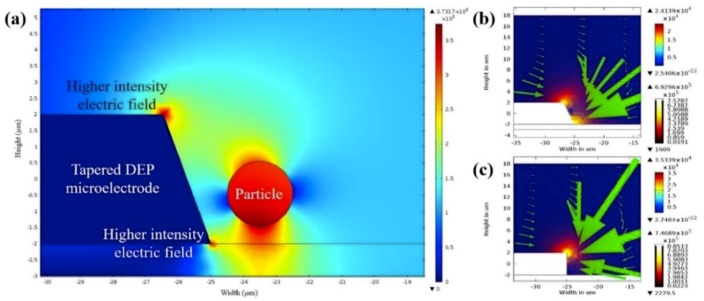
The FEM analysis of (**a**) the tapered microelectrodes at 70° electric field gradients, (**b**) the P_DEP_ at 70° and (**c**) the P_DEP_ at a 90° side wall angle.

The characterisation of lateral and vertical DEP forces (F_DEP_) was done by engineering a sidewall profile of the microelectrodes. Using FEM COMSOL, the first part of the characterisation of the tapered profile angle started from 5° to 90° with a step increment of 5°. The objective is to find two spots of high intensity electric field and further analyse the F_DEP_ distribution. Based on the FEM simulation result, the tapered DEP microelectrode profile angles at 70° generate two spots of high intensity electric field at the bottom and top edges of the sidewall of DEP microelectrodes. FEM simulation is used to validate the F_DEP_ distribution of two spots of high intensity electric field in analysing P_DEP_, the lateral attraction in y-axis, and N_DEP_, the vertical repulsion in z-axis, as shown in Figure 4a–c.

**Figure 5:**
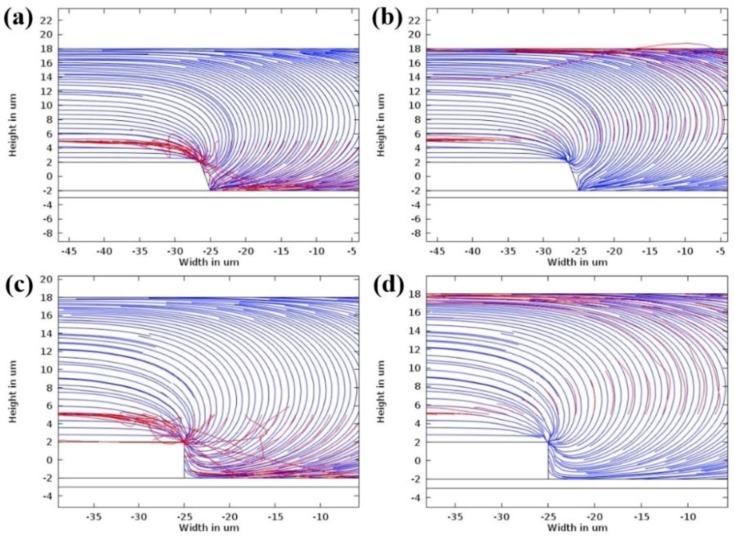
The FEM analysis of the particle trajectory for P_DEP_ and N_DEP_, (**a**,**b**) the tapered microelectrode 70° and (**c**,**d**) the straight-cut 90° microelectrodes.

The second part of FEM simulation involves the comparison on the positioning and stationing of particles between the tapered and straight-cut profile DEP microelectrodes. Based on the primary FEM simulation of the electric field gradient, the tapered profile DEP microelectrode shows two spots of higher intensity electric field compared to straight-cut profile DEP microelectrode with one spot of higher intensity electric field. Furthermore, analysis from the FEM simulation of P_DEP_ particles in the positioning and stationing of the tapered profile DEP microelectrodes starts at the bottom edge and final top edge of microelectrodes under P_DEP_. Meanwhile, the N_DEP_ particle positioning and stationing of the tapered profile DEP microelectrodes initially begins at the top edge and ends at the bottom edge before the repulsion between two DEP microelectrodes occurs as shown in Figure 5a,b. Compared to the straight cut, the particle positioning and stationing occurs only at the top edge of microelectrodes under P_DEP_ and N_DEP_. 

The implementation of tapered DEP microelectrodes is further described as an illusion of the pull or push to the particle trajectory using two hands. Particle manipulation and separation are firmly driven using two hands compared to the straight-cut microelectrodes that used one hand at the top edge of the microelectrodes. The pull or push using one hand created a large particle-tripping area known as vortex and ripple in particle manipulation and separation as shown in Figure 5c,d compared to the pull and push of particles using two steady hands from the edges of upper and lower tapered microelectrodes. This factor proves that the attraction or repulsion of two-handled particles produces an even and directed F_DEP_. This is one of the advantages of producing two high intensity electric field areas. This factor is important as it can cause F_DEP_ to be feasible to direct and drive particle mobility to the top surface and between two tapered microelectrodes.

### 3.2. Polarisation Factor

**Figure 6:**
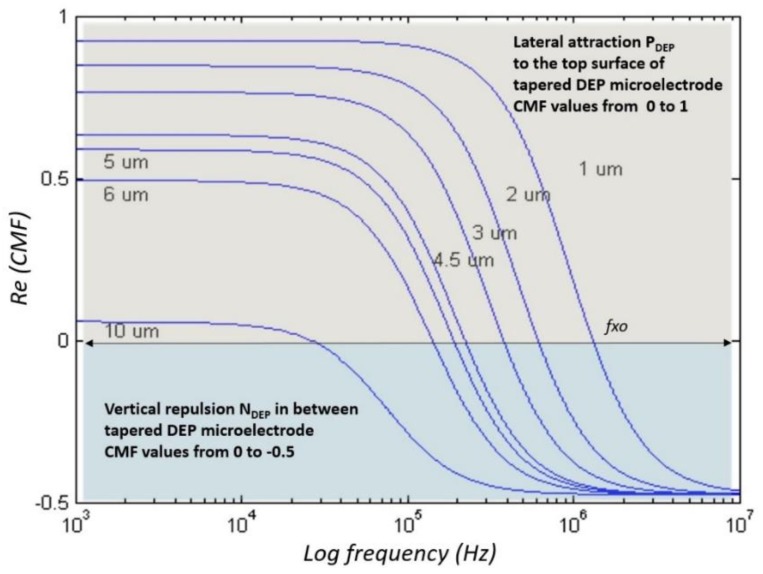
The polarisation factor of the Clausius–Mossotti factor (CMF) for Ps.

The DEP manipulation and separation processes are based on dynamic dielectric value changes between particles and mediums subjected to the frequency of the alternating current input applied. Based on the physical and chemical properties of each particle suspended in a liquid medium, the Clausius–Mossotti factor (CMF) is translated into dielectric properties to define a unique identification via *f_xo_* and a frequency adjustment *fadj*. Meanwhile, the Maxwell Wagner interfacial polarisation equation is used to formulate the frequency dependency of particles and mediums related to the permittivity and conductivity value as shown in Figure 6.

Analytical analyses of the polarisation factor are used to estimate the working input frequency response for each particle based on its size that reflects dielectric properties. Figure 6 displays the polarisation factor for polystyrene (Ps) with different particle sizes ranging from 1 to 10 μm. In the case of 10 μm, the CMF polarisation factor started with a P_DEP_ at low frequency at high magnitude by referring to the x- and y-axes of the plot, respectively. Further, an increase in the input frequency under P_DEP_ is done to reduce the magnitude until *fxo*. After that, Ps 10 μm is exposed to N_DEP_ but at low magnitudes until a further increment in input frequency at higher frequency is observed. 

The FEM simulation is used to validate the application of the tapered DEP microelectrode with two spots of high intensity electric field. Subsequently, the analytical polarisation factor is used to determine the input frequency response based on the particles and medium dielectric properties. The implementation of tapered DEP microelectrodes in an FEA simulation based on the defined polarisation factor results in the positioning and stationing laterally at the y-axis and vertically at the z-axis in hydrodynamic laminar flow at the x-axis. This results in the manipulation and separation of particles at two different locations at the top surface and between two tapered DEP microelectrodes. Consequently, the FEA simulation and polarisation factor finding is an experimental work realisation by the fabrication of tapered DEP microelectrodes. 

### 3.3. Fabrication

**Figure 7:**
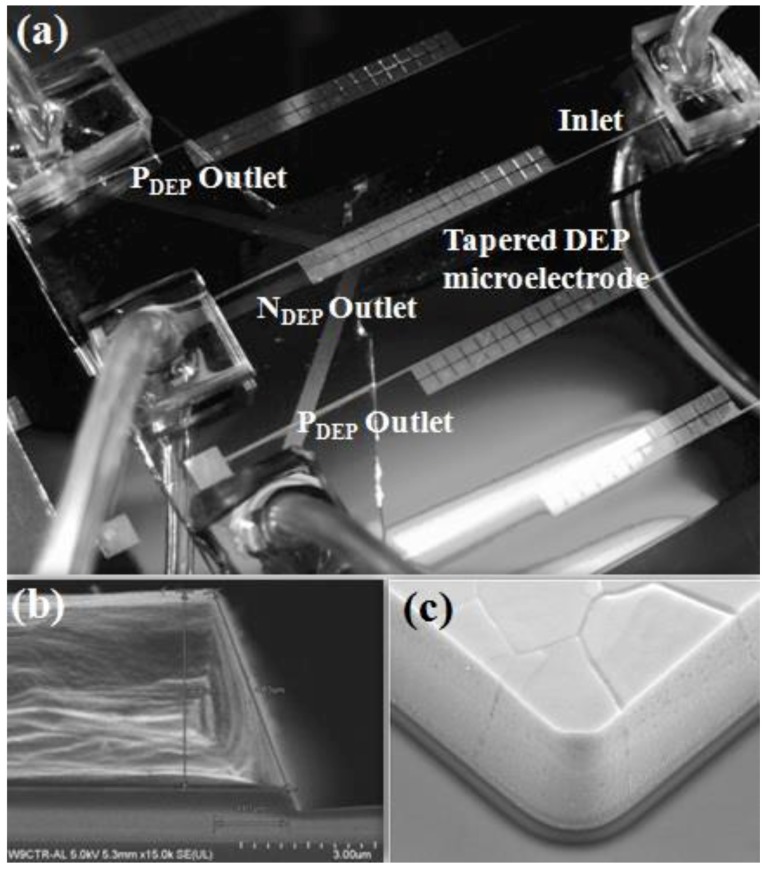
The tapered DEP microelectrode: (**a**) integration with one inlet and three outlet microchannels of (**b**) the side and (**c**) tilt views of the tapered profile angle 70°.

The fabrication process of tapered DEP microelectrodes uses CMOS-compatible process methods [40,41]. The critical part of the fabrication of tapered profile angle 70° requires an etching process characterisation and optimisation in controlling the sidewall etching profile angle [40]. The microfluidic microchannel is fabricated using the Polydimethylsiloxane (PDMS) hybrid fabrication method [40,41]. The configuration with a single inlet and three outlets is illustrated in Figure 7. The exposed P_DEP_ determined the position and station of target particles at the top surface of the tapered DEP microelectrodes. The hydrodynamics laminar flow yielded a P_DEP_ collected at the end left and right output outlet at the microfluidic microchannel. In contrast, the exposed N_DEP_ determines the position and station of the target particles between tapered the DEP microelectrodes. The hydrodynamics laminar flow yields a N_DEP_ collected at the end middle output outlet of the microfluidic microchannel.

## 4. Discussion

The invention of the tapered DEP microelectrodes is tested with three different conditions and samples. Analyses of the manipulation and separation capabilities by the lateral and vertical direction involved engineered particles and biological cells. These began with the manipulation process by positioning and stationing one type of engineered particle Ps further for the separation process of two different types of biological cells, red blood cells (RBC) and platelets and finally of three different sizes of engineered particle Ps. 

### 4.1. Manipulating Engineered Particle Ps Sizes of 10 μm

**Figure 8:**
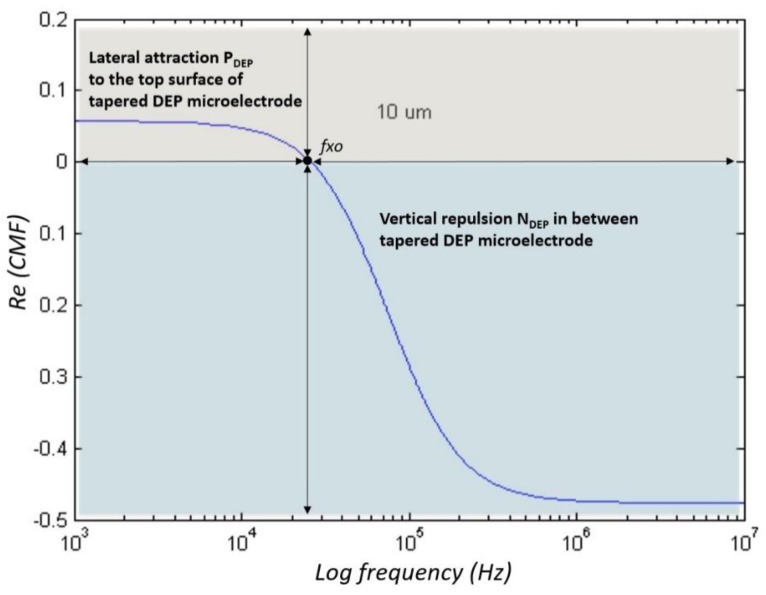
The polarisation factor of CMF for Ps 10 µm.

**Figure 9:**
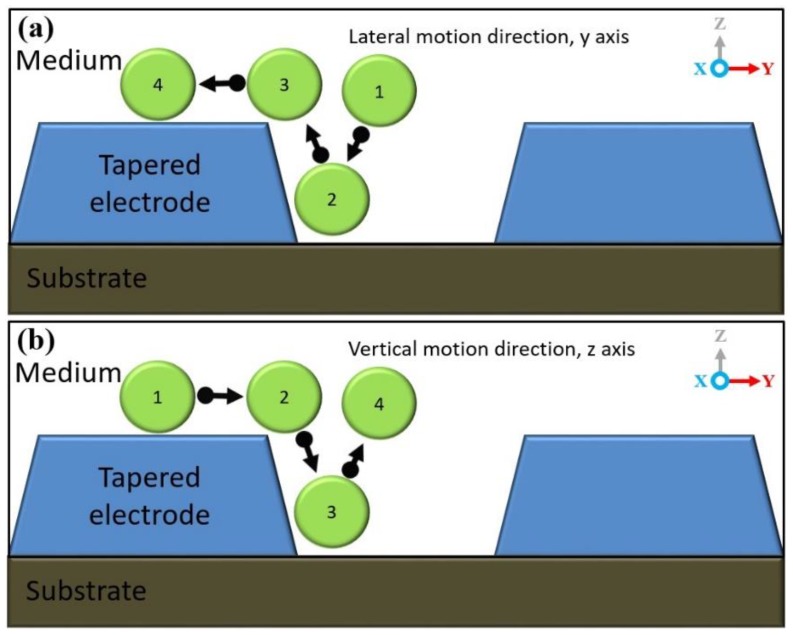
(**a**) At the input frequency 10 KHz, 10 μm Ps attracted laterally in the y-axis, P_DEP_, and (**b**) at the input frequency 2 MHz, 10 μm Ps vertically repelled in the z-axis, N_DEP_.

Based on Matlab analytic, analyses of the CMF polarisation factor for Ps are further verified by experimental work. Figure 8 shows the polarisation factor of CMF for Ps 10 µm. The experimental work observation on the manipulation of 10 μm Ps showed two different types of DEP response. At low frequencies, before *fxo* was P_DEP_. The positioning started from between two microelectrodes that laterally attracted at the y-axis towards stationing at the top surface of the microelectrodes as shown in Figure 9a. From low frequencies to *fxo*, the magnitude of lateral attraction is decreased with the intersection at CMF = 0. At this point, the polarisations of P_DEP_ and N_DEP_ are equal to the F_DEP_ force; thus, there is no force to drive the particles due to it being at the static point. The increased input frequency after *fxo* is N_DEP_ where the positioning starts from the top surface of the microelectrodes that vertically repels at the z-axis towards stationing between two microelectrodes as shown in Figure 9b. The magnitude of N_DEP_ and the vertical repulsion increases as input frequency increases, which is inverted to P_DEP._

**Figure 10:**
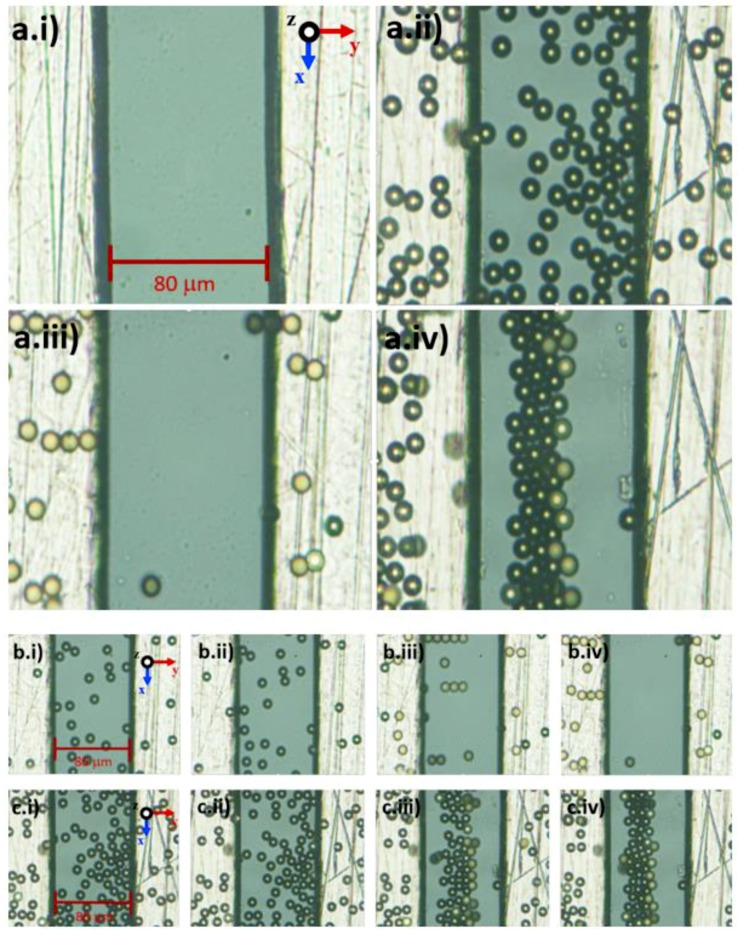
(**a**) The movement and direction of Ps in different conditions: (a.i) Di ionised water only, (a.ii) Di ionised water and Ps, (a.iii) P_DEP_ and (a.iv) N_DEP._ (**b**) At the input frequency 10 KHz, 10 μm Ps laterally attracted in the y-axis, P_DEP_: (b.i) 2 s, (b.ii) 5 s, (b.iii) 8 s and (b.iv) 10 s. (**c**) At the input frequency 2 MHz, 10 μm Ps vertically repelled in the z-axis, N_DEP_: (c.i) 2 s, (c.ii) 5 s, (c.iii) 8 s and (c.iv) 10 s.

Based on the experimental results as shown in Figure 10, the efficiency of manipulation using tapered DEP microelectrodes is observed to examine their ability to produce two higher intensity electric field areas at the bottom and top edges of the microelectrodes. The experimental observation of Ps shows that the manipulation process is based on P_DEP_, the lateral attraction at the y-axis, and N_DEP_, the vertical repulsion at the z-axis.

### 4.2. Separation of Two Different Types of Biological Cells, RBC and Platelets

**Figure 11:**
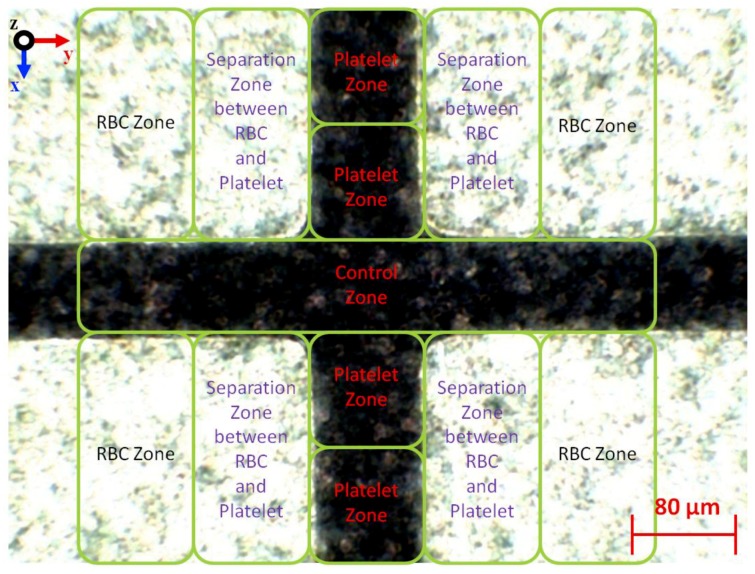
The separation, RBC and platelet zones.

Based on the manipulation capability of one type of Ps, further experimental work is done on the separation of two types of biological cells of RBC and platelets. Since both lateral attraction and vertical repulsion occurred parallel in positioning and stationing, this study defines several zones as shown in Figure 11. For RBC, the positioning started between two tapered DEP microelectrodes and end up stationing at the top surface of microelectrodes in the RBC zone via the separation zone between RBC and platelets due to the P_DEP_ lateral attraction force. For platelets, the positioning starts from the top surface of the microelectrodes and end up stationing between two tapered DEP microelectrodes at the platelet zone. This results in the yield of separation collected at the end array of tapered DEP microelectrodes using a designated one inlet with trio outlet microchannel. The RBC is collected at the left and right outlets of the microchannels due to the lateral attraction accumulated at the top surface of microelectrodes known as the RBC zone. Meanwhile, the platelets are collected at the middle outlet of the microchannels due to the vertical repulsion accumulated between two tapered DEP microelectrodes known as the platelet zone. 

**Figure 12:**
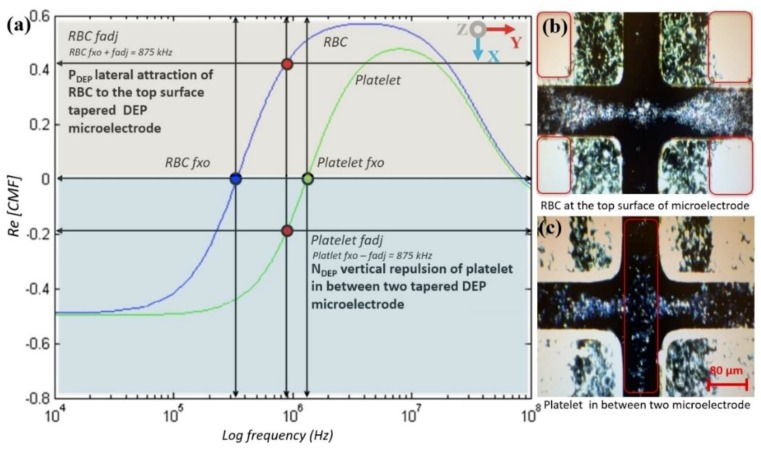
The CMF polarisation factor of the RBC and platelet zones.

Separation at two different locations at the top surface of microelectrodes and between two microelectrodes was based on the manipulating polarisation factor, CMF, of RBC and platelets. The unique identification of the physical and chemical dielectric properties for RBC and platelets is presented in different *f_xo_*. Different RBC and platelets *f_xo_* were used as references to find a new *fadj* for the separating of RBC and platelets as shown in Figure 12. The *fadj* presents different polarisation factors, the RBC in P_DEP_ lateral attraction to the top microelectrode surfaces and platelets in N_DEP_ vertical repulsion between microelectrodes. 

**Figure 13:**
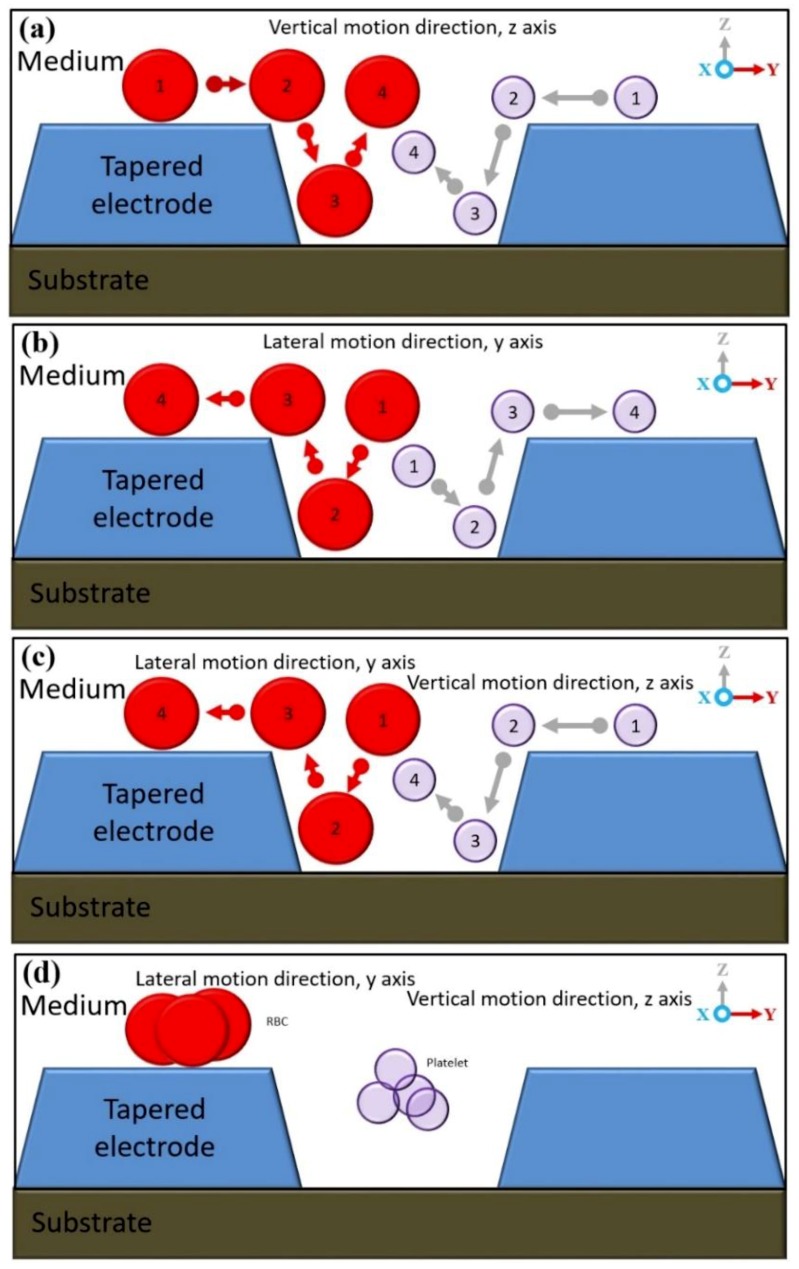
The separation of RBC and platelets: (**a**) Sequencing of the separation at the low input frequency region where both RBC and platelets were exposed to N_DEP_ vertical repulsion at the z-axis; (**b**) at the high input frequency region where RBC and platelets were exposed to P_DEP_ lateral attraction at y-axis; (**c**) within the *fxo* input frequency region where *fadj* produced different polarisations between the RBC and platelets, P_DEP_ lateral attraction at the y-axis and N_DEP_ vertical repulsion at the z-axis, respectively; and (**d**) separation at two different locations, which are RBC at the top surface microelectrodes and platelets between two microelectrodes.

The manipulation of the input frequency value is applied using the CMF polarisation factor based on the tapered DEP microelectrodes, which generates versatile manipulations with references of *fxo*. In the case of RBC and platelets, three different regions of input frequency are classified as low within *fxo* and high frequencies. At low input frequency previously applied within the *fxo* input frequency region, RBC and platelets were exposed to N_DEP_ vertical repulsion at the z-axis as shown in Figure 13a. At a high input frequency applied within the *fxo* input frequency region, RBC and platelets are exposed to P_DEP_ lateral attraction at the y-axis as shown in Figure 13b. Thus, at low and high input frequencies, both RBC and platelets were exposed to the same polarisation in N_DEP_ vertical repulsion at the z-axis and P_DEP_ lateral attraction at the y-axis, respectively. However, in the region within *fxo*, the *fadj* determined the separation of RBC and platelets subjected to the reference *fxo* of RBC and platelets. Therefore, *fadj is* used to produce different polarisations between RBC and platelets as shown in Figure 13c. Results from the separation of RBC and platelets at two different locations at the top surface microelectrode and between two microelectrodes are observed. Later, the result was collected at the end of array tapered DEP microelectrodes as shown in Figure 13d.

### 4.3. Separation of Three Different Sizes of Ps 1 μm, 3 μm and 10 μm

**Figure 14:**
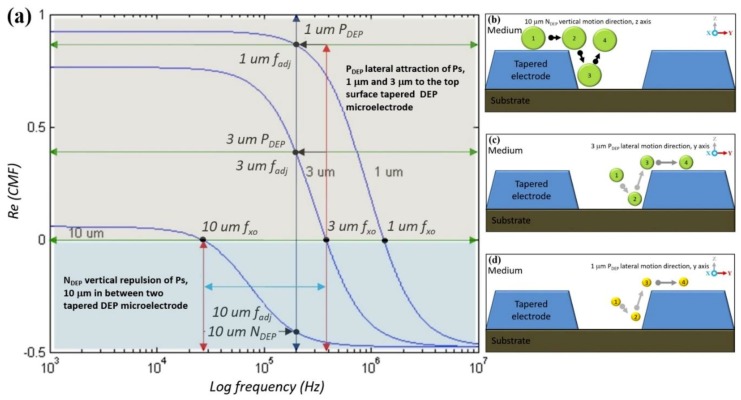
(**a**) The CMF polarisation factor and positioning and stationing of (**b**) 1 μm, (**c**) 3 μm and (**d**) 10 μm Ps.

The tapered DEP microelectrodes separation experimental study of the three different sized Ps is based on the CMF polarisation factor formulated from the dielectric properties model between the Ps and the medium for a mixture of 10 μm, 3 μm and 1 μm. The uutilisation of the mean of the midpoint *fxo* value between 10 μm and 3 μm of Ps yields a new and optimum input frequency separation as shown in Figure 14a. The new and optimum *fadj* value for the largest 10 μm Ps is driven by the N_DEP_ force, the vertical repulsion at the z-axis from two high intensity electric field areas. This results in the positioning of scattered 10 μm Ps to stationing at the region of interest between two tapered microelectrodes as shown in Figure 14b. Simultaneously, the smaller and smallest Ps of 3 μm and 1 μm are driven by the P_DEP_ force, the lateral attraction at the y-axis to two high intensity electric field areas. This causes the positioning of scattered 3 μm and 1 μm Ps to stationing at the region out of interest at the top surface tapered microelectrodes as shown in Figure 14c,d.

Figure 14b–d illustrates the sequence of the separation process for the mixtures of 10 μm, 3 μm and 1 μm for three different sized Ps. The output of separation was divided into groups. Collected at the middle outlet of the microchannel was 10 μm driven by the N_DEP_ force, the vertical repulsion at the z-axis between two tapered microelectrodes. Meanwhile, collected at the left and right outlets of the microchannel were 3 μm and 1 μm driven by the P_DEP_ force, the lateral attraction at the y-axis of the top surface tapered microelectrodes. Both responses were based on the CMF polarisation factor and manipulation of the *fadj* value.

**Figure 15:**
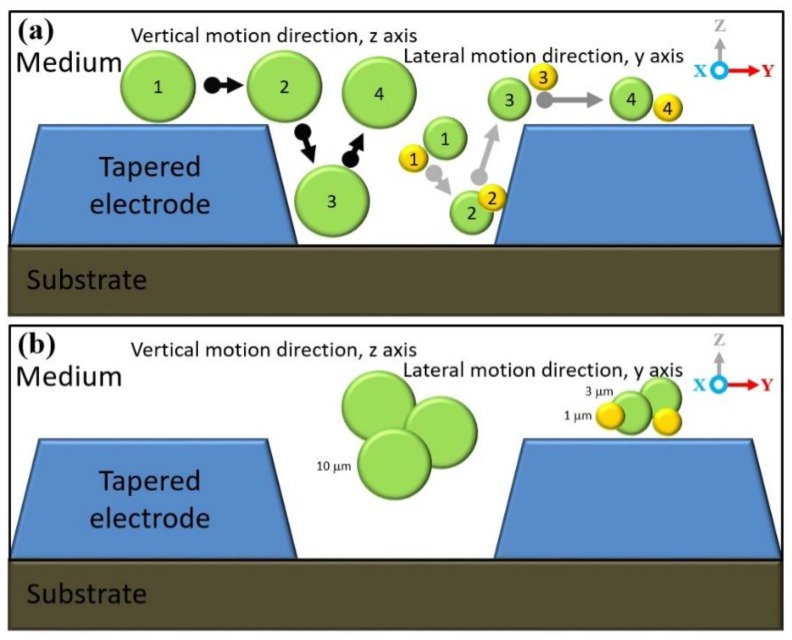
The side view separation sequence: (**a**) The separation steps of Ps 1 μm, 3 μm and 10 μm and (**b**) the separation at two different locations, Ps 1 and 3 μm, at the top surface and 10 μm between two tapered DEP microelectrodes.

**Figure 16:**
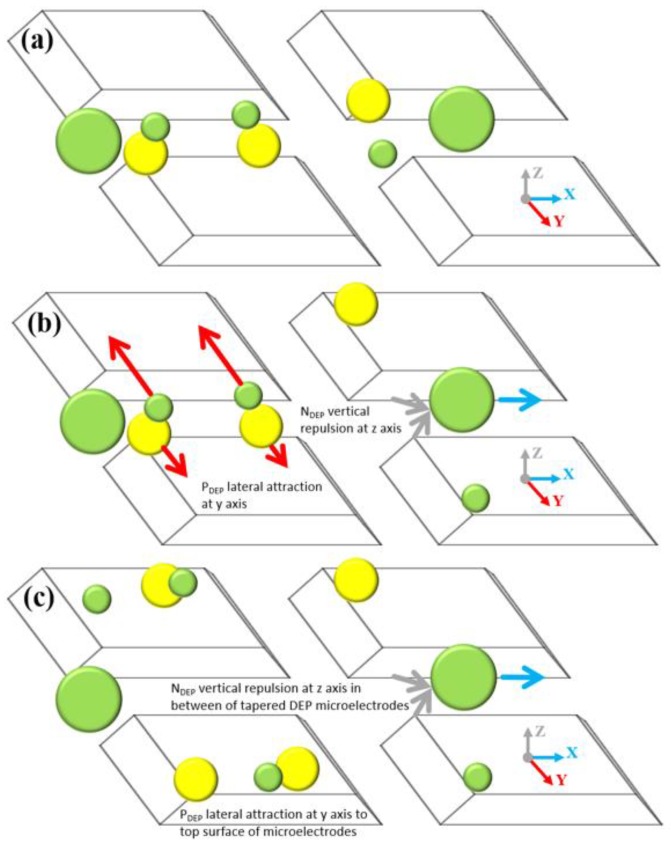
The tilt view sequence separation of 1 μm, 3 μm and 10 μm Ps: (**a**) The position before the input frequency is applied, (**b**) the separation sequence and (**c**) the separation yield.

Further experimental observations in the illustration side view are presented in Figure 15 with the details of the top view in Figure 16. Three different directions of Ps movements are recorded: First, the movement at the x-axis based on a continuous medium flow 1 μL/min, second, the positioning movement of 3 μm and 1 μm at the y-axis based on the horizontal attraction due to the P_DEP_ force and stationing at the top surface of the tapered microelectrodes, and third, the positioning movement of 10 μm at the z-axis based on the vertical repulsion due to the N_DEP_ force and stationing between two tapered microelectrodes.

The process separation of Ps 1 μm, 3 μm and 10 μm occurred due to a difference in the direction of the Ps movement. This results in the separation process at two different locations at the top surface or between tapered microelectrodes with the P_DEP_ lateral attraction at y-axis horizontally and the N_DEP_ vertical repulsion at the z-axis vertically. This versatile mechanism of the Ps position and station movement direction was due to the implementation of the tapered DEP microelectrodes with an angle of 70° and formulation of the *fadj* value. This justifies the advantages of the F_DEP_ exposure using tapered DEP microelectrodes. 

**Figure 17:**
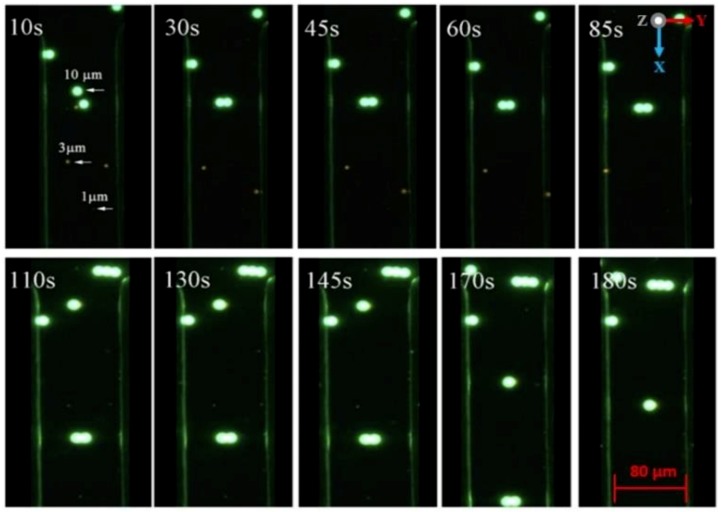
The sequencing of separation of 1 μm, 3 μm and 10 μm Ps at two different locations. 1 μm and 3 μm Ps were at the top surface microelectrodes while 10 μm Ps was between two microelectrodes.

Figure 17 depicts the experimental observations of the separation process sequence for the mixture of 1 μm, 3 μm and 10 μm. It was found that the actual experimental results were in line with the CMF polarisation factors with a proven capability of forming the P_DEP_ lateral attraction at the y-axis and the N_DEP_ vertical repulsion at the x-axis by producing two higher intensity electric field spots.

## 5. Summary and future perspectives

There are two types of DEP microelectrode sidewall profiles that expose the particles to the non-uniform electric field. The conventional configuration is the straight-cut profile [42,43,44] while innovative designs are based on the tapered DEP microelectrodes. In this review, we critically analysed microelectrode configurations and operating strategies and presented the opportunity for further enhancing the DEP capability in selective detection and rapid manipulation applications. The tapered DEP microelectrodes capability, the versatile mechanism by lateral attraction and vertical repulsion are alternative solutions for improving the lateral attraction or vertical repulsion only. The application of tapered DEP microelectrodes is towards the separation at two different locations rather than the straight-cut profile at one location only with different magnitude of attraction or repulsion for separation applications. Inspired by two-hands manipulation, the right and left hand side in manipulating the target and untargeted in two different locations should enhance the DEP selectivity. This review discussed the work in manipulating one type of particle known as Ps 10 µm, the separation of two types of biological cells namely RBC and platelets as well as the separation of three types of particle, namely 10 µm, 3 µm and 1 µm. Experimental work observations show the manipulation evidently at two different locations, which were at the top surface and between two tapered DEP microelectrodes. The critical and crucial points are during the applications involving biomolecules, for instance, in drug delivery and development in artificial organs. Particularly, mimicking human kidneys filtration requires a precision method. Thus, the combination of lateral attraction and vertical repulsion has been proposed to be an alternative solution instead of lateral attraction or vertical repulsion only. The concept of tapered DEP microelectrodes manipulation is proven using two different hands to achieve two different locations simultaneously. Compared to the lateral attraction or vertical repulsion only, this method enhances the manipulation capability and controllability instead of lateral or vertical manipulation using only one hand. 

Since its inception, DEP studies utilise straight-cut microelectrodes that generate one spot of higher intensity electric field at the top edge of sidewall microelectrodes as shown in Table 1. However, from 2015, with the implementation of advanced miniaturisation of microfabrication process technology, the tapered DEP microelectrodes that generate two spots of higher intensity electric field at the bottom and top edge of microelectrode sidewall is introduced. The advantage of generating two spots of higher intensity electric field is that ∇*E* generates two spots of force sources at the bottom and top edge of tapered profile microelectrodes. The main intention is to firmly enhance the selective detection and rapid manipulation with the capability of two different location positionings and stationings. Firm selective detection and rapid manipulation are referred to the movement of particles by stages. For example, particles exposed to P_DEP_ by lateral attraction at the y-axis. Initially, particles at the bottom edge move to the top edge microelectrodes before stationing at the top surface of the tapered DEP microelectrodes. Designated particles are exposed to N_DEP_ by vertical repulsion at the z-axis. Originally, particles positioned at the top edge moved to the bottom edge of the microelectrodes before stationing between two tapered DEP microelectrodes. The capability of selective detection and rapid manipulation at two different locations by lateral and vertical occurred simultaneously. This results in the selective detection and rapid manipulation of target and untargeted particles at the top surface and between two tapered DEP microelectrodes. The position determination of target and untargeted particle is done using CMF polarisation factors of *fxo* and *fadj* defined to be in or out of regions of interest at two different stations of location ends. This study introduces the selective detection and rapid manipulation F_DEP_ by combining P_DEP_ and N_DEP_ simultaneously via the implementation of tapered profile DEP microelectrodes. Efficient selective detection and rapid manipulation have been carried out utilising versatile mechanism laterally and vertically positioning and stationing with optimum input frequency *fadj* related to *fxo*. The outcome of the implementation of the tapered DEP microelectrodes was the combination of two different force directions [40] fabricated with CMOS Compatible fabrication technology [41]. This further results in the positioning and stationing of target/untargeted particles at the regions of interest with higher selective detection and rapid manipulation. This mechanism is validated using different sizes of engineered particle Ps and different types of biological cells. Thus, this study proposes a method by establishing alternative option for specification that requires both lateral and vertical manipulations, which can be compared with vertical or lateral manipulation designated at one specific location with separation capabilities by different magnitudes. 

Since its inception, DEP studies utilise straight-cut microelectrodes that generate one spot of higher intensity electric field at the top edge of sidewall microelectrodes as shown in Table 1. However, from 2015, with the implementation of advanced miniaturisation of microfabrication process technology, the tapered DEP microelectrodes that generate two spots of higher intensity electric field at the bottom and top edge of microelectrode sidewall is introduced. The advantage of generating two spots of higher intensity electric field is that ∇*E* generates two spots of force sources at the bottom and top edge of tapered profile microelectrodes. The main intention is to firmly enhance the selective detection and rapid manipulation with the capability of two different location positionings and stationings. Firm selective detection and rapid manipulation are referred to the movement of particles by stages. For example, particles exposed to P_DEP_ by lateral attraction at the y-axis. Initially, particles at the bottom edge move to the top edge microelectrodes before stationing at the top surface of the tapered DEP microelectrodes. Designated particles are exposed to N_DEP_ by vertical repulsion at the z-axis. Originally, particles positioned at the top edge moved to the bottom edge of the microelectrodes before stationing between two tapered DEP microelectrodes. The capability of selective detection and rapid manipulation at two different locations by lateral and vertical occurred simultaneously. This results in the selective detection and rapid manipulation of target and untargeted particles at the top surface and between two tapered DEP microelectrodes. The position determination of target and untargeted particle is done using CMF polarisation factors of *fxo* and *fadj* defined to be in or out of regions of interest at two different stations of location ends. This study introduces the selective detection and rapid manipulation F_DEP_ by combining P_DEP_ and N_DEP_ simultaneously via the implementation of tapered profile DEP microelectrodes. Efficient selective detection and rapid manipulation have been carried out utilising versatile mechanism laterally and vertically positioning and stationing with optimum input frequency *fadj* related to *fxo*. The outcome of the implementation of the tapered DEP microelectrodes was the combination of two different force directions [40] fabricated with CMOS Compatible fabrication technology [41]. This further results in the positioning and stationing of target/untargeted particles at the regions of interest with higher selective detection and rapid manipulation. This mechanism is validated using different sizes of engineered particle Ps and different types of biological cells. Thus, this study proposes a method by establishing alternative option for specification that requires both lateral and vertical manipulations, which can be compared with vertical or lateral manipulation designated at one specific location with separation capabilities by different magnitudes. 

DEP’s innovation integration with “lab-on-chip” technology is one of the best solutions available today. Improvement through the integration of CMOS-MEMS standardised fabrication technology for developing tapered DEP profile with microfluidic microchannel technology for medium-flow channels has succeeded in producing DEP labs-on-chips capable of performing particle selective detection and rapid manipulation processes at high efficiency. This high-efficiency definition explains the ability of tapered DEP microelectrodes to manipulate and separate particles in two directions in two different locations at one input frequency value applied. The particle movement is seen horizontally with the P_DEP_ attraction on the y-axis and vertically with the N_DEP_ push force on the z-axis using an input *fadj* based on *fxo* of target and untargeted particles. Therefore, it is considered a solution for the effects of major problems including physical contact with particles. In addition, it can reduce the contamination of particles and mediums due to direct contact and the addition of contaminated materials such as marker colour and magnetic. This review introduced and opened the ways for a rapid, label free, precision method to carry out selective detection and rapid manipulation of mixtures of red blood cells and platelets. Further potential applications are for protein, toxins, cancer cells and bacteria detection and removal. The determination of the lateral and vertical dielectrophoresis forces was done simultaneously through a new configuration of DEP microelectrodes arrays with a tapered side wall profile. Based on the analysis from this study, two directions of motion have been established by obtaining a new crossover frequency defined as adjustment frequency, *fadj*. The *fadj* was within the value between the *fxo* of RBC and platelets. The innovative design of tapered DEP microelectrodes arrays enabled F_DEP_ to selectively detect and rapidly manipulate RBC and platelets efficiently resulting in the identification and separation at two different locations. The selective detection and rapid manipulation at two different locations are at the top surface of microelectrodes and between two microelectrodes by DEP force [45,46,47,48].

These successes permit this work to introduce the dielectrophoretic sensor mechanism of *fxo* to detect different types of engineered particle and biological sample. In parallel, the dielectrophoretic actuator mechanism of *fadj* manipulates the target sample by the P_DEP_ and N_DEP_ forces. This proves the concepts for developing tapered DEP microelectrodes for both selective detection and rapid manipulation utilising a dielectrophoretic sensor and actuator. In terms of future perspectives based on communication with Ronald Pethig via Researchgate, he wrote, “When it comes to isolating target cells from a cell mixture, it would be better to think in terms of capturing the unwanted cells at the microelectrodes and repelling the target cells into the flowing bulk medium, so as to reduce the possible harmful effects of a strong field gradient on the membrane. Best wishes to you and your work.” The application of tapered DEP microelectrodes as suggested by Pethig captures unwanted cells by P_DEP_ lateral attraction at the y axis to the top surface of the microelectrodes as well as N_DEP_ vertical repulsion at the z axis repelling the target cells into the flowing bulk medium in between two tapered DEP microelectrodes for collection. This versatile filtration is one of the potentials for a glomerular-based membrane in artificial kidneys development.

## 6. Conclusions

There are two types of DEP microelectrode profiles exposed the particles to the uniformity electric field, which are straight-cut and tapered DEP microelectrodes. Straight-cut profile microelectrodes have the ability to manipulate and separate particles in y- or z-axes only. The introduction to tapered DEP microelectrode applications enabled the selective detection and rapid manipulation in y- and z-axes subjected to x-axis is a fluid flow path. Combining the selective detection and rapid manipulation processes in y- and z-axes served as the main advantage of applying tapered DEP microelectrodes. Two spot intensities of higher electric fields at the bottom edge and top edge of tapered DEP microelectrodes facilitated simultaneous selective detection with rapid manipulation laterally and vertically, which is one of DEP technology application improvement techniques. Tapered DEP microelectrodes are capable to perform the selective detection and rapid manipulation. Optimum F_DEP_, either P_DEP_ or N_DEP_, is utilised due to the construction of two spots of high intensity electric field areas. This proof of concept is useful in developing tapered DEP microelectrodes for both selective detection and rapid manipulation utilising dielectrophoretic mechanism as sensor and actuator. 

Dielectrophoresis has been proven capable for selective detection and rapid manipulation in microfluidic systems. The ability of combination of both lateral at the y-axis and vertical at the z-axis motion is desirable as it allows the enhancement of sensitive, selective and rapid method particularly in medical applications. This study introduces a method to produce and determine lateral and vertical motion using F_DEP_ in simultaneously utilising a new configuration of a tapered side wall profile, DEP microelectrode arrays with higher intensity electric field gradient at bottom and top edge of microelectrodes. This configuration enables two directions of motion to be established besides obtaining a new *fxo,* which was *fadj*, within the value between the crossover frequency of target and untargeted cells. The target particles can be biological cells in a mixture of cells and other particles or a mixture of cells with various physical and biological properties. The special design of tapered microelectrodes arrays enabled F_DEP_ to perform selective detection and rapid manipulation target species at the top surface and between two tapered DEP microelectrodes. This requirement provides a versatile filtration that can by potentially used for glomerular-based membranes in artificial kidney development solutions. Thus, it can be concluded that the implementation of tapered DEP microelectrodes had established a novel DEP platform for electrical driven with selective detection and rapid manipulation at high yield of efficiency capability.

## Figures and Tables

**Table 1:** The lateral, vertical and combination of lateral and vertical motion directions.

**F_DEP_, P_DEP_ and N_DEP_ by** **Lateral Attraction at the Y-Axis**	**Description**
[30,31,32,33]	Mechanism: One spot intensity electric field with P_DEP_, lateral attraction and N_DEP_, lateral repulsion at y-axis only Advantages: Simple electrode configuration Disadvantages: Manipulation and separation yielded at the y-axis only with different magnitudes. Relies on fluid flow fraction. Requires lots of microelectrode structures and a long fluid flow channel resulting in a time-consuming approach.
**F_DEP_, P_DEP_ and N_DEP_ by** **vertical repulsion at the z-axis**	**Description**
[34,35,36,37,38,39]	Mechanism: One spot intensity electric field with P_DEP_, vertical attraction and N_DEP_, vertical repulsion at z-axis only Advantages: Simple electrode configuration Disadvantages: Manipulation and separation yielded at the z-axis only with different magnitudes. Relies on fluid flow fraction. Requires lots of microelectrode structures and a long fluid flow channel resulting in a time-consuming approach.
**F_DEP_, P_DEP_ and N_DEP_ by** **lateral attraction at the y-axis and vertical repulsion at the x-axis**	**Description**
Present [40]	Mechanism: Two spots of intensity electric field generating the combination of P_DEP_, lateral attraction at y-axis and N_DEP,_ vertical repulsion at z-axis Advantages: Rapid and selective for manipulation and separation, which yielded at two different locations at the y- and z-axes, at the top surface and between two microelectrodes Disadvantages: Complex microfabrication process
